# Quality Thresholds for Angiogenesis Under Acoustic Manipulation in Engineered Vascular Tissues

**DOI:** 10.1002/advs.76219

**Published:** 2026-06-23

**Authors:** Oscar O'Dwyer Lancaster‐Jones, Russell Quinn, Niloofar Khoshdel Rad, Kilian Paul, Andrea Frank, Daniela F. Duarte Campos

**Affiliations:** ^1^ Bioprinting and Tissue Engineering Group Center of Molecular Biology of Heidelberg University (ZMBH) Heidelberg University Heidelberg Germany

**Keywords:** angiogenesis, biomedical engineering, extracellular matrix, gene expression, in vitro, tissue engineering

## Abstract

Tissue engineering can be accomplished in a plethora of ways, ranging from direct deposition of cells onto scaffolds and chips to contactless volumetric printing of 3D structures. Recent studies have examined the feasibility of using acoustic manipulation of cells in vitro and in situ, although these studies have not yet identified the critical quality requirements to create complex tissues. To this end, we present a methodology for generating and evaluating acoustically patterned 3D vascular tissue for biomedical studies. Over a period of 7 days, tissues were able to generate self‐assembling vasculature, deposit a self‐secreted extracellular matrix network, and demonstrate changes in angiogenic gene expression in response to acoustic patterning. These behaviors demonstrated a positive correlation between the quality of alignment and associated vascular structure following acoustic manipulation, suggesting a relationship between patterning and tissue development. These findings identify necessary requirements for creating self‐assembling vasculature in vitro, accounting for biological and physical parameters.

## Graphical abstract

Defined biological and physical quality thresholds achieved through acoustic manipulation regulate cellular alignment and spatial organization, promoting self‐assembling vasculature, extracellular matrix deposition, and angiogenic responses, thereby establishing critical acoustic patterning requirements for engineered vascular tissue development in vitro.

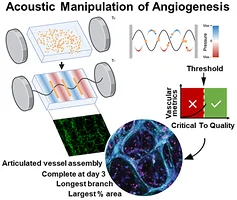

## 1 Introduction

Engineered tissues have become an important corner stone in the development of advanced 3D models which are capable of improved physiological relevance for disease and developmental studies. A number of tissue engineering techniques have been developed to provide such systems, ranging from traditional tissue casting, advanced bioprinting approaches, and dynamic culture in bioreactors of varying scales [1, 2, 3, 4, 5, 6, 7, 8, 9, 10, 11, 12, 13, 14, 15]. Each method has benefits and drawbacks that should be considered when developing a tissue construction workflow.

Much like native tissues, engineered tissues have high cell densities and large burdens for nutrients and gasses such as oxygen [10, 16]. While these needs can be safely met in traditional 2D cell culture, complex 3D structures are subject to limitations in the diffusion limit of oxygen (100–200 microns), and other nutrients [6, 10, 13, 16]. There currently exist two main strategies to create patent and perfusable vasculature in engineered tissues: incorporation of the bioink into a microfluidic chamber, which is subjected to fluid pressure, and transplantation of cell‐laden hydrogels in vivo [1, 17, 18, 19]. The most accessible of these two techniques is microfluidics, as the chips are relatively easy to work with and have associated technical support provided by established vendors. The generation of patent micro vessels in chips is also a popular approach for modelling tissue angiogenesis, and can accommodate both larger diameter vessels by pre‐seeding large channels with endothelial cells prior to perfusion, or smaller networks by providing a stable flow rate across a cell‐laden 3D hydrogel with varying cell complexity [3, 9]. Naturally, microfluidic chips do have some drawbacks, notably the technical challenges of extracting chip‐based tissues for replacement therapies at scale [8, 12]. It is possible, however to transplant premade tissue patches, or perform in situ fabrication techniques where a suitably sized tissue construct can be implanted. Approaches which include extrusion‐based bioprinting, drop‐on‐demand, and volumetric printing have become increasingly popular for these purposes. That is not to say however, that we are limited to these examples.

Regardless of which method is used, establishment of the vasculature network is a crucial first step when developing tissues in vitro, in situ, or in vivo. Unfortunately, not all model systems have the flexibility to be created exclusively in microfluidic chips or transplanted into animals/patients. In particular, complex engineered tissues for disease modeling and drug testing often need to be run in high‐throughput formats, which often require well plate or transwell plate culture protocols, which are rarely compatible with perfusion [20]. In response to this, the development of self‐assembling vasculature, which does not need external forces to organize, has become a vital area of interest in the field of tissue engineering [12, 13, 19]. Such model systems generated have proven valuable in understanding tissue dynamics and pathological mechanisms in an accessible in vitro setting [11, 13].

A recent novel method to fabricate engineered tissue is the use of acoustic waves to pattern non‐adherent cells in medium and hydrogel [1, 2, 17, 18, 19, 21, 22]. The technique works by applying a vibration, which results in either an acoustic radiation force or acoustic streaming. The most direct method is the use of acoustic emitting piezo electric transducer with matched wavelengths (standing waves) travelling through a medium, which can trap the cells in place at the node points (locations where pressure fluctuations are more stable). The propagation of the wave can be along the surface, this is called Surface Acoustic Waves (SAW), for example, using an Interdigital Transducer (IDT) [18]. Another method is to use the entire surface of a piezo, which is defined as Bulk Acoustic Waves (BAW) [22]. Studies have applied acoustics to work on various types of cells/tissues, such as muscle [23], heart [24, 25], cartilage [26, 27], and vasculature [1, 2, 17, 18, 19, 21, 28, 29].

For vasculature in particular, Comeau et al., and Kang et al., have developed acoustic systems capable of patterning cells in the sub‐dermal region of mice using primary and iPSC‐derived endothelial cells [1, 18]. They were able to pattern sub‐dermally injected bioinks, which were incorporated into the microvasculature structure of the mice. By co‐opting the native supporting cells found in the mouse tissue with the implanted endothelial (and adipose‐derived stem cells in the case of Kang et al.,), they were able to generate patent vasculature [1, 18]. Contactless methods of tissue patterning also offer the advantage of being minimally invasive when compared to other methods such as drop on demand and extrusion‐based bioprinting [7].

Regardless of methodology, engineered tissues are designed to recapitulate relatively complex 3D behavior, which is not typically achievable in conventional 2D culture systems [8, 9, 10, 11, 13]. To that end, it is important to determine the Critical To Quality (CTQ) parameters for functionality for a given behavior/structure of interest. For patterned engineered tissue, one way to gain insight is the Voronoi Tessellation evaluation, which provides a metric for cell density or cell‐cell relative position [30]. Whilst this evaluation is a very good metric, it alone cannot compare alignment and conformity of shapes, or be used as a single quality requirement metric for functional tissue. When manufacturing tissue with acoustics, the environment can cause imperfections due to wave refractions, phase shift, or acoustic streaming, and if the intent is to carry out these for patient treatment, it is important to consider what verification steps can be taken at the site of intervention or in the lab without much interference.

To these ends, we have developed a working protocol to generate patterned vascular tissue with acoustic manipulation, as well as an accompanying analysis method to help determine what are the CTQ parameters for acoustically manipulated vascular tissues in a 3D in vitro setting. The tissues generated with this protocol are capable of creating self‐assembled capillary networks, secreting their own extracellular matrix (ECM), exhibiting mature phenotypes such as the co‐localization of perivascular cells with endothelial tubes, and forming patent hollow vasculature. Additionally, we observed acoustically driven changes in gene expression related to angiogenic growth and cell recruitment. In summary, we present a working protocol and associated tools for the creation of engineered vascular tissues. We also outline the procedure for the assessment of quality of alignment and conformity in engineered tissues in vitro and allude toward minimum requirements.

## 2 Results

### 2.1 Creation of Acoustic Platform and Co‐Culture Model

**FIGURE 1:**
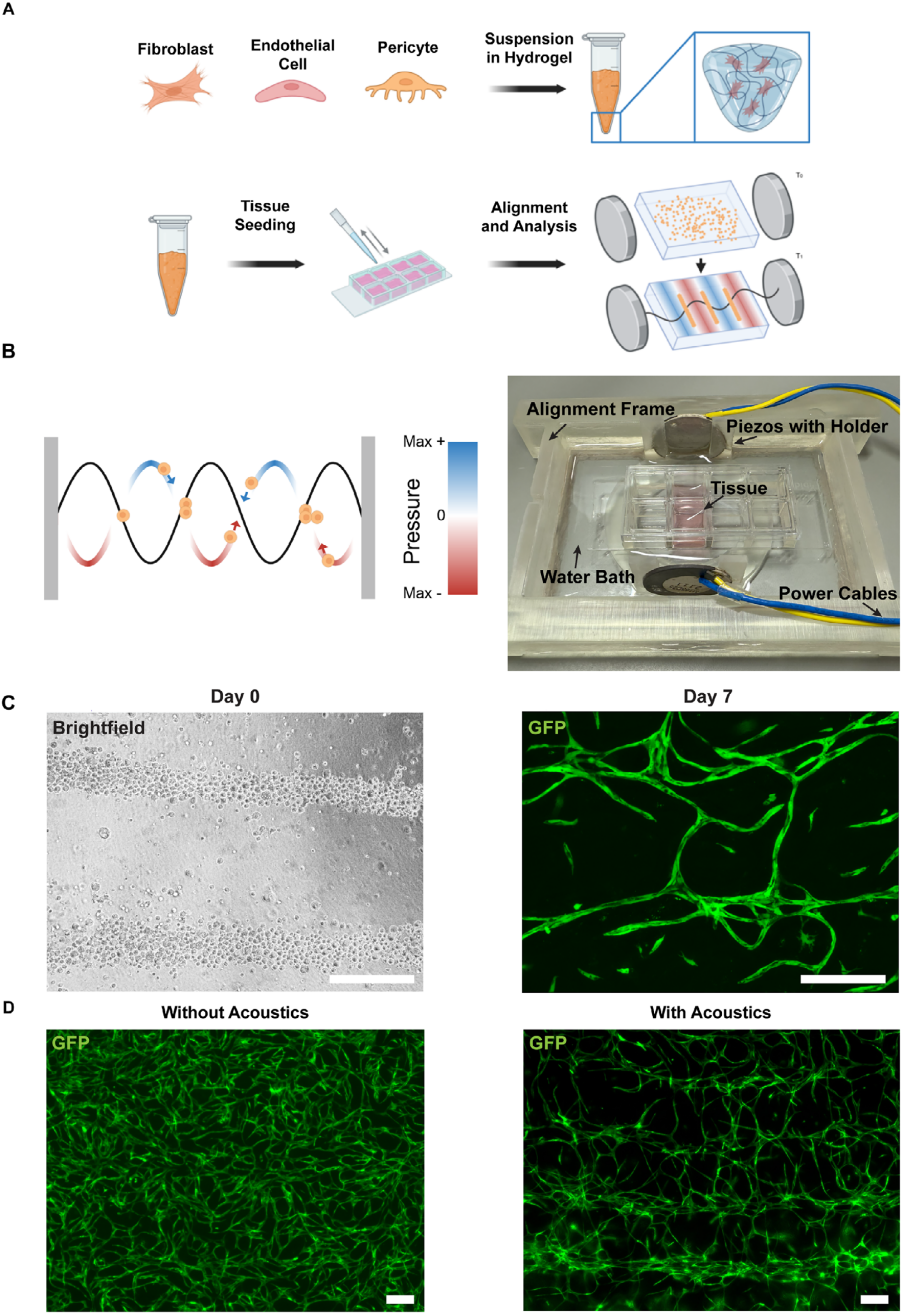
Development of self‐assembling vascular tissues with acoustic manipulation. (A) Schematic approach for the creation of aligned 3D vascular tissues. Cell types were suspended in hydrogel, and patterning was formed via acoustic manipulation after tissue seeding. Schematics were created in BioRender. Standing wave schematic of cell manipulation where pressure oscillations shift the cells into the node positions. (B) Benchtop acoustic manipulator holder for a multi‐chamber slide inside a platform with water. Scalebar = 10 mm. (C) Brightfield and GFP images (10X) of acoustically manipulated cells at day 0, and a matched GFP image with vessel formation at day 7. Scalebar = 300 µm. (D) GFP images (4X) of vessel formation after 7 days with and without acoustic manipulation Scalebar = 200 µm.

In order to develop our acoustically patterned tissues, we first needed to construct a platform to create standing waves (Figure 1A) and a corresponding 3D culture system that was compatible with this methodology. To this end, we fabricated a platform using a 3D resin printer to hold chambered culture slides, piezoelectric transducers, and a basin for water (Figure 1A). This platform was then connected to a signal generator and amplifier to generate the standing waves (Figure 1B). Thus, our method uses bulk acoustic waves. These waves provided sufficient pressure to manipulate the position of our cells towards the node points where the pressure was at its lowest (Figure 1B). This system was designed with our particular capabilities in mind, but is similar in principle to a number of other ultrasound systems [1, 17, 18, 19, 22, 23, 26].

With our apparatus assembled, we then needed to establish our 3D culture system. Based on current literature, we identified a fibrin‐based hydrogel system as the optimal choice for growing self‐assembling vasculature [13, 19]. This was due in part to being able to enzymatically control the polymerization rate of the fibrinogen to fibrin, which enabled us to “lock” the cell patterns into shape over a period of 7 min (Figure 1B,C, Video S1). To this end, we suspended a mixture of fibroblasts, endothelial cells, and pericytes in a fibrin solution, and proceeded to polymerize the bioink while subjecting the cells to acoustic waves (with a 1.66 MHz frequency). Two chambers with the same bioink were subjected to acoustic manipulation simultaneously. Chamber heating was minimal, and the piezo reached a maximum operating temperature of 28°C. This resulted in condensed cell bands with an average of 150 microns in width (Figure 1C,D). This resulted in a stable culture that was capable of producing confluent vasculature over the course of 7 days (Figure 1D).

### 2.2 Determining Quality of Alignment and Relevant Parameters

**FIGURE 2:**
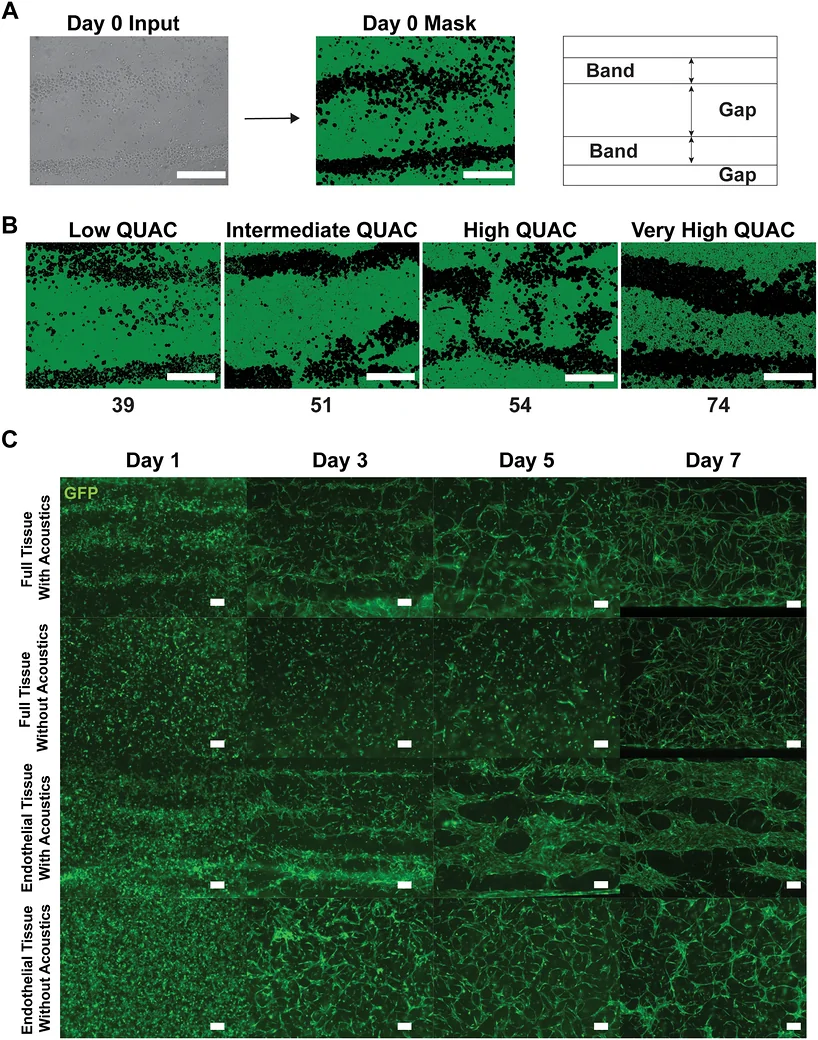
Assessment of cell position and progression of vascular tissue. (A) Representation of image processing. Brightfield images (10X) were taken at day 0, and processed to identify optical percentage density. Cells were then analyzed according per region and scored. Scalebar = 300 µm. (B) Processed images after acoustic manipulation and corresponding quality of alignment and conformity score. Images were processed at day 0. Scalebar = 300 µm. (C) GFP images of 3D tissue without or with acoustic manipulation over the course of 7 days. This was performed on both endothelial cell‐only tissues as well as Full Tissue. Images were taken with a 4X objective on an EVOS microscope. Scalebars = 200 µm.

To evaluate the acoustic cell manipulation, images of the cells after polymerization were taken and processed to determine the percentage area occupied by the cells (Figure 2A). A script was used to determine the density of the tissue and the area in the bands. Thus, a quality score was produced depending on how well the cell's position conformed to the bands (Figure 2B). Whilst the co‐cultured “Full Tissue” was capable of creating vascular networks over 7 days, the tissues with acoustic manipulation began forming vessel‐like structures as early as day 3 (Figure 2C). Likewise, when the endothelial cell‐only tissues were created, the acoustically manipulated variants seemed to organize earlier when compared to the control group.

### 2.3 Relationships Between Cell Clustering From Acoustic Manipulation and Vascular Variance

**FIGURE 3:**
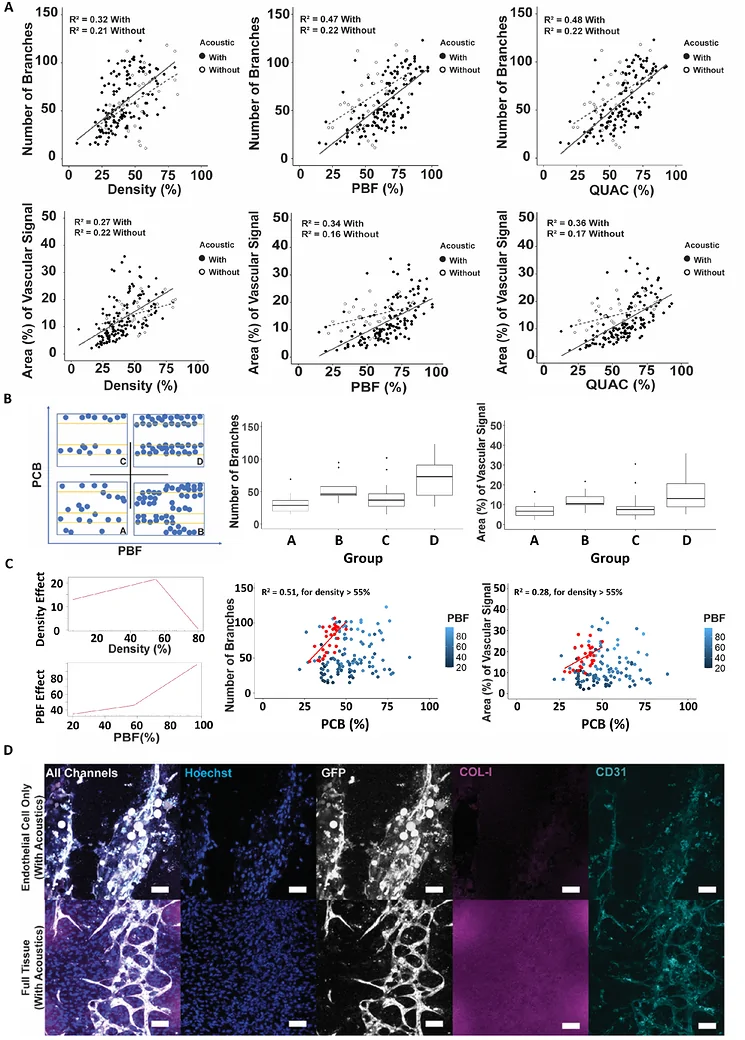
Assessment of vascular tissue. (A) Correlations between the number of branches and signal area percentage at day 7 for density, Percentage of Band Fill (PBF), and Quality of Alignment and Conformity (QUAC) with and without acoustic manipulation. Pearson correlation values are presented for each graph. With and without acoustic manipulation, n = 141, n = 27 respectively. (B) Number of branches and signal area percentage assessment at day 7 versus high and low patterning score for cells with acoustic manipulation with boxplots for density below 55% (n = 114). Schematic of group representation in relation to percentage of Cells in the Bands (PCB) and PBF. Group A for less than 60% PBF and group B for more than 60% PBF, both of these groups with PCB of less than 40%. Group C for less than 60% PBF and group D for more than 60% PBF, both of these groups with PCB more than 40%. (C) Piecewise breakpoints plot effect for density and PBF. Correlations for the number of branches and area for PCB (n = 141). Red dots, trend line, and Pearson correlation for density above 55%. Points below 55% density in the blue heatmap. (D) Immunostaining images for collagen type‐I (COL‐I, magenta), CD31 (CD31, cyan), endothelial cell GFP reporter (GFP, white), and Hoescht (blue) on aligned flatmount tissues of endothelial cell only (Top) and Full Tissues (Bottom). Images were taken with a 20X objective on a Leica SP5 confocal microscope. Tissues were 7 days old at the time of acquisition. Scalebar = 75 µm.

Images were matched for band formation (day 0) and vascular tissue (day 7). To explain the data, correlations were made for percentage cell density, Percentage of Band Fill (PBF), and the Quality of Alignment and Conformity (QUAC) for the number of branches and area (Figure 3A). Each of these evaluations, density, PBF, and QUAC, provided good correlations (R^2^) for the number of branches (0.32, 0.47, and 0.48 respectively, all *p* < 0.001) and area (0.27, 0.34, and 0.36 respectively, all *p* < 0.001) with acoustic manipulation. To determine the impact of adequately aligning to the expected band position as intended from the test conditions i.e. conforming to the area of the band and a repeating gap spacing of approx. 370 microns, the data was categorized into four groups by two parameters (Figure 3B). How well does it fit to the intended band location, which is the Percentage of Cells in the Bands (PCB), and the percentage of band fill. To determine the breakpoint, a piecewise evaluation was carried out for PBF and density (Figure 3B). Taking a threshold point above 60% (breakpoint 58% ± 6.7 SE, Figure 3C) percentage band fill, had no difference whether the cells were aligned in the intended band or not (i.e., below or above 40% PCB, Figure 3B, group B and D) for the number of branches and vasculature percentage area. No effect was determined from the multiple regression for PCB (*p* = 0.08) or the interaction of PCB and PBF (*p* = 0.08). From the suggested breakpoint of 55% ± 8.1 SE for density, a subset of the data had a positive correlation with PCB (Figure 3C). This indicated once a threshold of 55% density was reached, cell arrangement had an effect on the number of branches. Multiple regression analysis confirmed the interaction effect between density and PCB for the number of branches (*p* < 0.005), but not for area (*p* = 0.5). The resulting full tissues demonstrated articulated vessel assembly, complete with ECM deposition (Figure 3D). This was not the case with endothelial cell‐only tissues, which would not generate vascular structures regardless of acoustics (Figure 3D). We also determined that while the number of branchpoints and total area covered by vasculature were not significantly different between acoustic and non‐acoustic conditions, we did observe increases in the length of branchpoints (Figure S2A–D). Such branch elongation has been seen as a positive indication of vascular development, often as a result of ECM stiffening and perivascular recruitment around nascent vessels [31]. This is hypothesized to allow new vessels to form and reinforce their lumens prior to vascular expansion [31, 32].

### 2.4 Structural Features of Self Assembling Vascular Tissues

Based on image quantification, we have been able to establish a working baseline for what factors contribute to optimal vessel‐like structure formation, and resulting influences on vascular development. Based on other contemporary work in the field, this is typically where most studies are completed [17, 21]. This does present a knowledge gap in whether complex tissue structures in 3D culture systems in a manner which is consistent with the results gleaned from basic analysis [10, 13, 33]. To further expand upon this subject, we prioritized examining the colocalization of fibroblasts, pericytes, and endothelial cells in mature vessels, identifying the presence of extracellular and intracellular structures via ECM deposition and cytoskeletal arrangement, and finally the development of hollow inner lumens without the presence of peristatic pressure.

**FIGURE 4:**
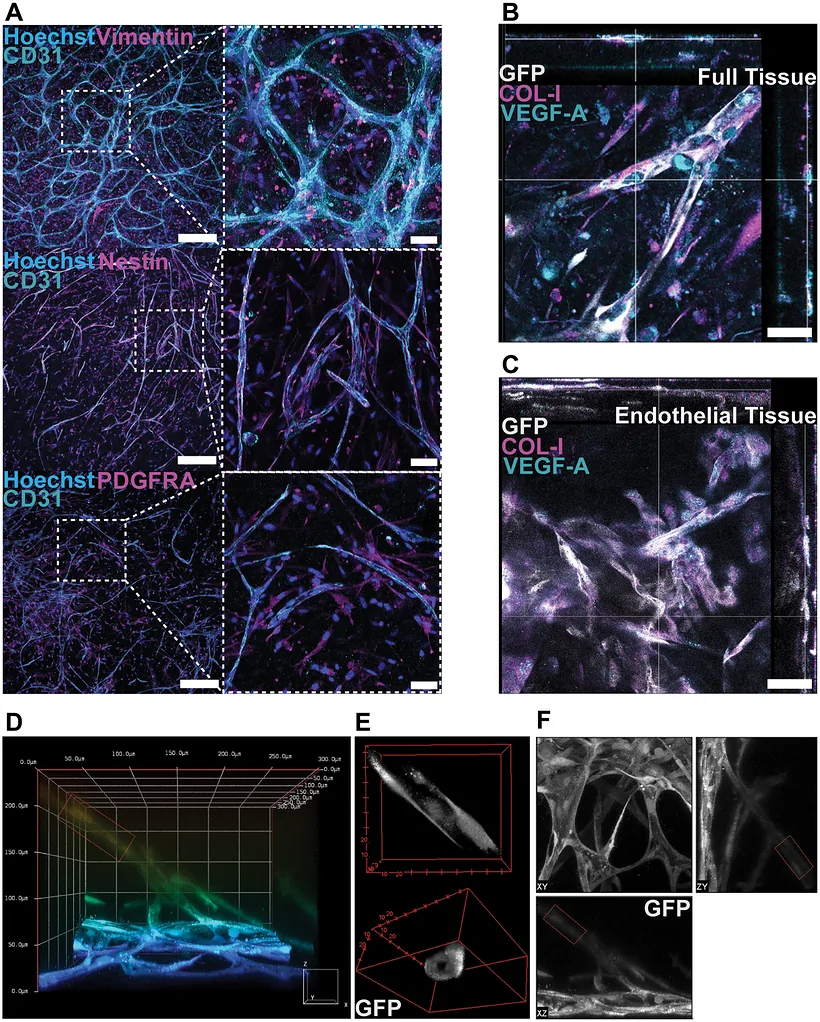
Evaluation of mature structural phenotypes and vasculature in tissues with acoustics. (A) Maximum intensity projection immunostaining images for fibroblast identity markers (PDGFRA, Vimentin, magenta), endothelial cell markers (CD31, cyan), pericyte markers (Nestin, Magenta) and Hoescht (blue) on flatmount samples of Full Tissues with acoustic manipulation. Images were taken with a 10X objective on a Leica Stellaris confocal microscope. Tissues were 7 days old at the time of acquisition. Scalebar = 200 µm. Magnified views of the boxed regions in panel A are shown on the right. Scalebar = 50 µm. (B) Immunostaining images for collagen type‐I (COL‐I, magenta), Vascular Endothelial Growth Factor A (VEGF‐A, cyan), and endothelial cell GFP reporter (GFP, white) on acoustically manipulated Full Tissues. Images were taken with a 63X objective on a Leica SP8 confocal microscope. Tissues were 7 days old at the time of acquisition. Scalebar = 50 µm. (C) Immunostaining Images for collagen type‐I (COL‐I, magenta), Vascular Endothelial Growth Factor A (VEGF‐A, cyan), and endothelial cell GFP reporter (GFP, white) on acoustically manipulated endothelial‐only tissues. Images were taken with a 63X objective on a Leica SP8 confocal microscope. Tissues were 7 days old at the time of acquisition. Scalebar = 50 µm. (D) 3D reconstruction of acoustically manipulated vascular tissue. Immunostaining images for endothelial cell GFP reporter (GFP) on Full Tissue. Tissues were 7 days old at the time of acquisition. XZ projection of the volumetric image data with the height of the sample colour‐coded (bottom blue‐cyan‐green‐yellow‐red‐top) over 240 µm in Z. (E) Texture‐based 3D renderings of the volume outlined in D and F (red boxes) are shown as a side view (top) and spatially rotated to see the cross‐section of the tubular structure (bottom). The metric dimensions of the volume are displayed. (F) Maximum intensity projections along the XY (top left), ZY (top right), and XZ (bottom left).

First, we were able to document clear colocalization between endothelial cells and pericytes in mature 7‐day old vasculature via confocal imaging (Figure 4A). Additionally, we observed widespread fibroblast and pericyte expansion in our cultures, with partial overlaps with CD31 vasculature (Figure 4A). Indeed, it would seem without the presence of fibroblasts and pericytes, the endothelial‐only tissues were not capable of secreting sufficiently organized ECM space to promote vasculature. The structural support provided by the co‐culture was also sufficient to generate hollow inner capillary lumens, which were not otherwise present in the endothelial cell‐only tissue (Figure 4B–D, Video S2, Video S3). Such behavior has been well documented previously, highlighting the requirement of collagen type 1 secreted by fibroblasts for the formation of vascular structures [13, 34]. The condensed cell bands resulted in vasculature with an approximately 100 microns in height (Figure 4D). The resulting actin cytoskeletal arrangements is also noticeably different between the co‐culture and endothelial cell only conditions (Figure S3). It is important to note that these behaviors are consistent between with and without acoustic‐manipulated tissues. In other words, the act of patterning our tissues with acoustic waves is not sufficient to disrupt this critical behavior, thereby maintaining its utility as a methodology for vascular tissue engineering.

### 2.5 Acoustic Manipulation of Cells Influences Angiogenic Gene Expression

**FIGURE 5:**
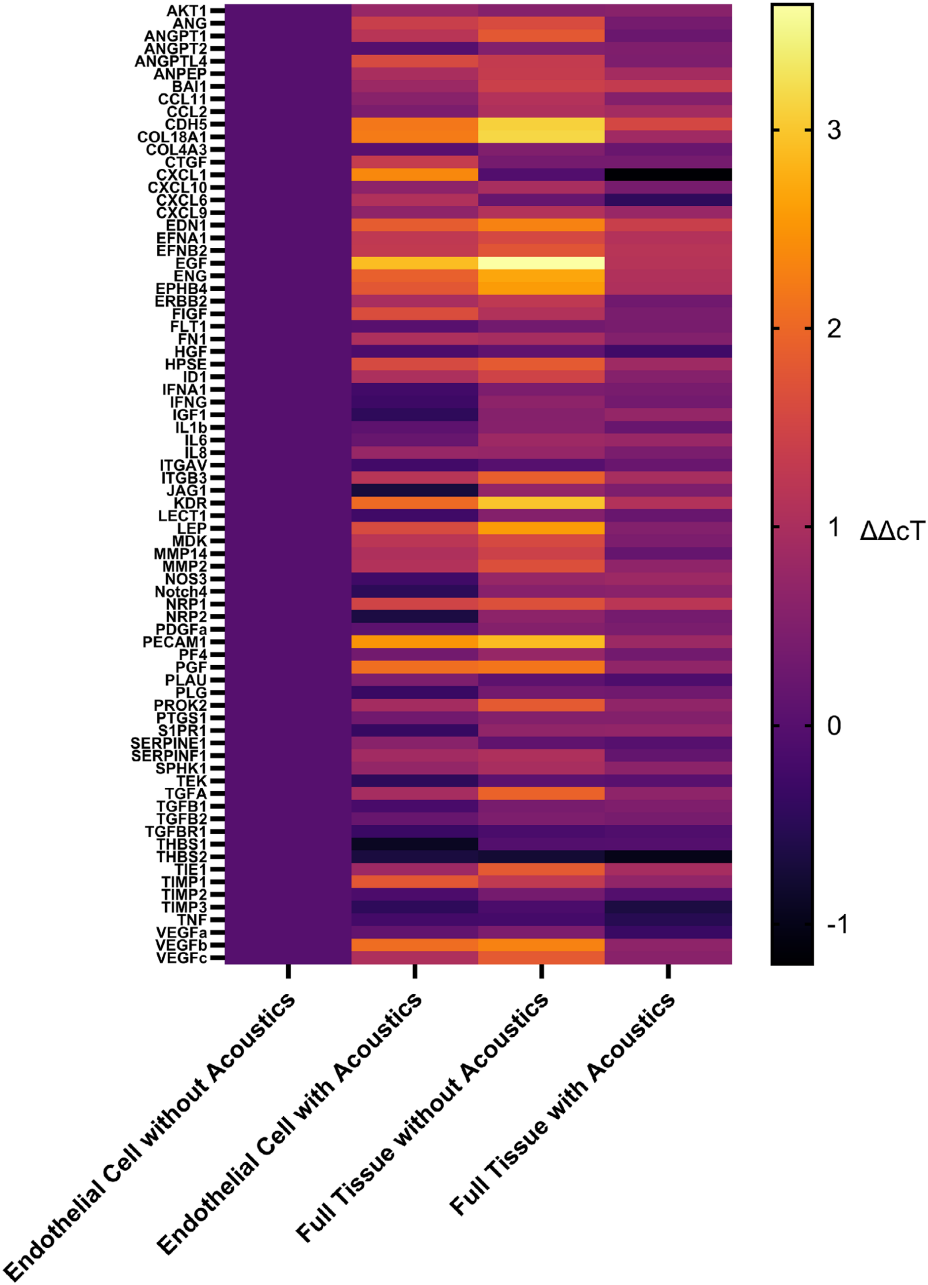
Observable changes in angiogenic gene expression via acoustic forces. Angiogenic PCR gene expression panel comparing tissues with and without acoustic manipulation across endothelial cell‐only and Full Tissue samples. Heat map displays mean fold change for ΔΔCT, corresponding to the color gradient on the right. Data were analyzed with Two Way ANOVA, with Tukey's and Sidak's multiple Comparisons Test. Full statistical relationships can be found in the extended supplement. Samples came from individual biological replicates with an n = 4.

Previous studies have established that engineered 3D tissues benefit greatly from synergistic crosstalk between resident cell types for regulating ECM deposition, angiogenesis, inflammatory response, and many other cell signaling pathways [4, 13, 35]. Of particular interest to us was the possibility of mechanically transduced gene expression resulting from our 3D environment and associated acoustic forces [36, 37, 38]. Indeed, recent work by Kumeta et al., demonstrated that prolonged ultrasonic stimulation in 2D adipocyte culture was sufficient to change numerous cell signaling pathways [38]. This happened to include angiogenic signaling. To this end, we compared the gene expression profiles via quantitative PCR analysis between our 4 tissue groups. Endothelial Cell (EC) only tissues without acoustic manipulation served as a baseline for ΔΔCT values alongside housekeeping genes: Actin‐Beta (ACTN), Beta‐2‐microglobulin (B2M), Glyceraldehyde‐3‐Phosphate dehydrogenase (GAPDH). Hypoxanthine phosphoribosyltransferase 1(HPRT1), and Ribosomal protein, large, P0 (RPLP0). We observed a number of resulting changes in expression across all angiogenic‐related genes in response to acoustics and co‐culture conditions, with many genes seeing 1–2 fold increases, and decreases in acoustically manipulated tissues (Figure 5A). The most notable changes observed in acoustically manipulated tissues were CDH5, COL‐18A1, EGF, KDR, LEP, TIMP family, and VEGF family. Acoustic manipulation was sufficient to cause a twofold increase in CDH5 expression in EC‐only cultures, but only demonstrated trend‐based changes in Full Tissue conditions. CDH5 (better known as VE‐Cadherin) is a vital protein for the establishment of nascent vasculature and blood barriers [39]. Similarly, genes such as EGF, KDR, CXCL1, PECAM1, and VEGF‐b are all known to be potent enhancers of angiogenesis [40, 41, 42, 43] and saw corresponding 1–2 fold increases when compared to EC only tissues without acoustics. ECM remodeling, also a critical regulator of angiogenesis, saw significant increases in deposition of collagen subtypes such as Collagen 18A1 [44]. While this single cell type behavior was interesting and supported the prior findings of Kumeta et al., the expression data for the Full Tissues diverged in terms of behavior when comparing acoustically manipulated Full Tissues to their counterparts [38]. For one, acoustically manipulated Full Tissues demonstrated decreased angiogenic expression when compared to non‐manipulated tissues (Figure 5). Genes such as Collagen 18A1, EGF, KDR, LEP, and PECAM1 all saw decreases in the Full Tissue with acoustics. This could be due to a number of factors, including how fibroblasts and pericytes respond individually to acoustics. In fact, it is not uncommon for fibroblasts and pericytes to negatively regulate angiogenesis under certain conditions or in response to the correct stimuli [45, 46, 47]. Observing such complex, opposing behaviors in co‐cultured 3D engineered tissues is well documented, as the added complexity of the tissue environment can stimulate more advanced regulatory behaviors [48, 49, 50]. While our PCR panel was limited in scope compared to the sequencing data presented in Kumeta et al., our results also demonstrate that acoustic manipulation of cells is sufficient to alter angiogenic gene expression in both 3D mono‐culture and co‐culture, although optimized culture conditions and bioink formulations are necessary for angiogenic growth [38].

## 3 Discussion

Our acoustically manipulated vascular tissue model demonstrates a reproducible methodology to create complex multi‐cell‐type tissues with advanced structures in a contactless manner. A key finding was that vasculature was more prominent, based on the number of branches and area, when the percentage band fill was greater than 70%, and a disruption to the intended pattern didn't play much of a role below 55% density. Our data suggests that angiogenic behavior is enhanced by said acoustic manipulation, and correlates positively with mature vascular structure, including ECM deposition, and the self‐organization of capillaries with hollow inner lumens without the need of incorporated perfusion via peristatic pump or animal implantation. Our results suggest that vascular tissue formation is enhanced by the presence of pericytes and fibroblasts in the culture, in addition to the included media supplements, which promote dynamic instability for the purposes of angiogenesis and the formation of ECM structure [36, 37, 38]. These components are further affected by acoustic manipulation, with related changes in angiogenic, ECM, and inflammatory signaling. Overall, a QUAC higher than 60% is a good baseline requirement for vasculature subjected to acoustic manipulation, in comparison to tissues without acoustic manipulation. The findings we present provide an improved foundation for defined vascular tissue co‐culture regarding acoustic manipulation, and mechanistic inferences towards acoustically mediated gene expression.

Understanding the margin of error to generate acoustically manipulated vascular tissues and whether there is a difference between poor‐ and high‐quality patterned areas to create vasculature has been a key driver in this research. To establish CTQ metrics in our tissues, we first evaluated the parameters that influence vasculature. Density provided a good initial basis, but the percentage of band fill had a better correlation for the acoustically manipulated tissues. However, these metrics alone are not enough to compare bands that are either partially broken and slightly shifted or away from the intended node position with a smaller/larger gap. Thus, first we compared the expected pattern location using a metric of PCB along with high and low percentage of band fill. Second, we derived an equation that combines the density, percentage band fill, and gap distance to infer a single metric that describes the quality of the tissue. When evaluating the data for shorter and wider gaps, the QUAC equation shows a minor improvement to percentage of band fill (Figure S1B,C), however this is not considered a significant improvement based on the variation, and the gap spacing had a minor effect. No difference was also demonstrated by Garvin et al., for gap spacing of 375 and 750 microns [21]. Taken together, they allowed us to establish a baseline for band quality, which could be later compared with vascular tissue growth and maturation on day 7. It was interesting to find a density threshold where below 55% the main factor was percentage band fill, and after 55% density, the driver for the amount of vasculature is suggested to be the cell‐cell relative position, followed by percentage band fill. If we compare to a Voronoi Tessellation evaluation, the metrics we provided seemed to account for the inherent variation (Figure S1A, Table S1). Evaluations like ours can be used in more complex tissue formations, whether using acoustic manipulation or not, for specified shapes where Voronoi Tessellations may not be the most suitable evaluation [51, 52]. Our co‐culture protocol demonstrated that vasculature can form with or without acoustic manipulation (Figure S2), but is positively influenced by the density of cells in a given area as other studies have shown [21, 53]. From a CTQ perspective, it is not necessary to utilize a single metric to define the tissue. Percentage band fill on its own is acceptable, and other parameters can be added to the matrix as required based on the intended use.

Studies into the practical applications of acoustic patterning have been successfully described in previous publications for well over a decade [1, 2, 21, 22, 25, 28, 54]. These observations are typically from single‐cell‐type in vitro studies, which prioritize biocompatibility and utility of systems with the cell types and culture format used as opposed to detailed structural and behavioral characterization [1, 2, 21, 28]. More recent work has improved upon this, focusing on multi‐cell‐type co‐culture systems which are capable of generating more complex secondary structures, such as aligned fibrils found in cardiac and cartilage tissue niches [23, 24, 26]. The work of Armstrong et al., has been particular insightful into the construction of patterned/aligned cellular filaments during post patterning culture periods [23]. All of these studies provided qualitative and quantitative analyses to determine how the process of acoustic patterning contributed towards tissue formation, going beyond basic qualitative image analysis and linking mechanistic behavior to observed phenotypes.

Armstrong, Comeau, Kang, and others were capable of constructing single or dual‐cell‐type systems for establishing patterned tissues in vitro and in situ, as previously outlined. While such systems are well designed, they lack the ability to generate microvascular networks and native ECM, which are necessary for larger‐scale tissues [4, 6, 13, 55]. To this end, our initial approach focuses on advanced morphological features seen in self‐assembling co‐cultured vasculature, such as recruitment of perivascular cells, which sheathe established capillaries, and help mediate dynamic instability associated with tip formation and sprouting through the TIE‐2 signaling pathway [54, 56]. This is a hallmark of mature capillaries, and suggests synergistic co‐stimulation of angiogenic growth via the TIE‐2 signaling pathway as well as general supportive behavior associated with endothelial cell/pericyte interactions [31, 32, 57]. The inclusion of fibroblasts in this mixture was also key, as we observed native ECM deposition (Collagen I) and rearrangement of actin networks in our co‐culture when compared to the endothelial cell‐only tissues [58, 59]. These fibroblasts appear to play a key role in organizing the local ECM environment via secretion and organization of collagen, as well as the cytoskeletal arrangement of the tissue. While unable to perfuse our tissues, we were able to demonstrate hollow inner lumens for our co‐cultured capillaries. Self‐organization of such structures have been previously demonstrated to support larger‐scale tissues, and are a crucial component in creating all manner of thicker tissues which exceed the diffusion limit of oxygen (100–200 microns) [10, 13, 16]. As described in the results, our tissues can range from 80–170 microns in thickness, which would require hollow microvascular networks. Our findings, consistent with previous studies, demonstrate a working method to develop patterned vascular networks capable of efficiently diffusing oxygen and other gases throughout a tissue greater than 100 microns in thickness.

One particularly interesting observation was the potential change in gene expression in tissues that were subjected to acoustic radiation. While various kinds of mechanical/physical stimuli are known to alter gene expression both in vivo and in vitro, the impact of acoustic forces in this behavior are not fully understood [5, 37, 38]. Recent studies, such as Kumeta et al., show that acoustic forces are capable of changing gene expression in monocultured 2D cells in a multi‐factorial manner [38]. In their study, they determined it was possible to alter the expression of specific genes to modulate the differentiation of adipocytes, although it was different to our work with regards to frequency, intensity, cell types, culture format, and type of wave generation used (e.g., standing vs. travelling). Although our study was conducted in 3D, we also observed noticeable changes in the expression of genes related to angiogenesis. It should be noted, however that these changes could also result from increased cell density and changes to hydrostatic pressure (as outlined in Kumeta et al.,) [38]. Our results also suggest that there may be a complex interplay in pro and anti‐angiogenic behaviors in response to acoustic patterning, and that these changes may be driven in a cell‐type‐specific manner [45, 46, 47]. This presents a potentially interesting avenue of study, which would require cell‐level resolution techniques, including single‐cell RNA sequencing to untangle. In summary, it is quite possible that acoustically driven changes in gene expression directly contribute to the development of our vascular tissue, although further study is required to eliminate the possibility of secondary influences unique to the 3D culture environment.

This model system and associated analysis tools are not without limitations. Acoustic streaming, to some extent distorted bands and affected cell populations between bands. Further complexities may arise from different cell sizes. The acoustic radiation force may keep larger cells in nodes, whereas smaller cells could drift off due to streaming leading to higher variation (see Figure S4). Our fibrin‐based hydrogel had variable polymerization rates as well, and would vary within a 1–5 min time span from experiment to experiment. This included the time required to mix and dispense our samples prior to acoustic manipulation of the hydrogels. Our live imaging techniques also relied on traditional microscopy to capture images of 3D tissues. 2D images may not also cover 3D dimensional densities appropriately. As our tissues were over 100 microns in thickness, there would be some cell signals outside the range of the objective's working distance, potentially causing limited variance in our measurements. That said, such variance would not be enough to significantly alter the results, as our correlation values were found to be satisfactory. Localized vascular measurements were not decoupled from combined gene expression evaluations and tissue‐wide structural analysis. To strengthen the gap effect analysis, a parametric evaluation with different frequencies (node spacing) would be ideal. The suggested equations and values for comparison are for guidance, as they have not been tested with other forms of 3D tissue patterning, such as continuous *z*‐direction 3D patterning or extrusion‐based bioprinting, for example. The findings we present may not cover data beyond the range we tested. In future studies, it may be possible to perform multi‐cell labeling prior to tissue casting in order to better differentiate cell clustering variability and effect. Additionally, a high‐content confocal system with large working distances, integrated environment chamber, and fast scanning speed would be appropriate for generating volumetric tissue data.

In terms of applications for this type of tissue patterning, it is comparable to more common techniques such as extrusion and inkjet printing. That said, it also comes with its own inherent benefits and challenges. Where the acoustic patterning method may be most useful, is in the fields of high‐throughput tissue generation and non‐invasive in situ printing [1, 7, 18, 20]. As covered earlier in this article, Comeau et al., were successful in performing non‐invasive tissue patterning in the sub‐dermal region of mice using acoustic patterning. Such techniques, if developed properly, could stand to enhance surgical options for tissue replacement therapies through minimally invasive procedures. Additionally, methods such as extrusion‐based printing are limited to creating one tissue at a time, while a properly aligned acoustic emitter system is capable of patterning numerous tissues simultaneously. If this system were to be scaled up to high‐throughput well formats and organ‐on‐a‐chip systems (such as devices from Emulate or Mimetas), new custom‐built arrays could provide an exponential decrease in the time required to create engineered tissues [59]. Needless to say, this would provide an efficient system for High‐Throughput Screening (HTS) drug discovery applications.

Taken together, this acoustically manipulated vascular tissue protocol provides new insights into patterning techniques and their effects on vasculogenic growth and tissue maturation. By using an easily constructed and accessible system, it is possible to generate confluent vasculature in 3D, which self‐assembles into a mature network capable of sustaining hollow tubes and native ECM. With a stable vascular bed in place, this model can be adapted to a number of 3D tissue constructs and in vivo systems that benefit from vascularization [2, 3, 6, 9, 10, 13, 16, 21, 22, 58]. Finally, the suggested metrics emphasize the importance of CTQ thinking for engineered tissues and their fabrication process.

## 4 Methods

### 4.1 Assembly of Alignment Apparatus

The holder was 3D printed (Saturn 2, ELEGOO) with Blu V2 clear resin (Siraya Tech). This holds a chamber slide (µ‐Slide 8 Well chamber, Ibidi, Cat# 80807) between two piezoelectric transducers (MD165D25, Midas, Farnell). Piezoelectric transducers were attached to the holder with super glue (45415, UHU). The distance between the piezos was 50 mm, and the ibidi slide was placed centrally with a gap between the piezo of 12.25 mm. Using the supplier data, chamber dimensions are 9.4 × 10.7 mm (w x l). To cover the chamber size, the piezo had a direct open central window of 16 mm. As 150 µl hydrogel was dispensed in the chamber, this equates to an approx height of 1.5 mm. A platform was also 3D printed to provide a basin for the sample, including the water surrounding the holder and slide for wave transfer. The water basin was filled with a minimum of 40 mL of water with the aim to cover the height of the hydrogel tissue, this volume equates to an approx. height of 2 mm. The benchtop system utilizes an MFG 2230 signal generator (GW Instek) connected to an amplifier (LZY‐22, Minicircuits). Impedance evaluation (Analog Discovery 2 and Impedance Analyzer, Diligent) of piezoelectric transducers identified their resonating frequencies at 1.66 MHz (see Figure S5).

### 4.2 Tissue Culture Media

Fibroblasts and pericytes were cultured in FibroLife Basal Medium + growth factor kit (Cell Systems, Cat# LL‐0001). Endothelial cells were cultured in VascuLife Basal Medium + growth factor kit (Cell Systems, Cat# LL‐0005). Vascular Tissue medium was comprised of a 1:1 mixture of VascuLife Basal Medium + growth factor kit and FibroLife Basal Medium + growth factor kit. Vascular Tissue Medium also contained 85 ng/mL Vascular Endothelial Growth Factor (VEGF) (Biotechne, Cat # 293‐VE‐050/CF), 0.075 U/mL Aprotinin (Merck, Cat # A1153‐100MG), and 100 ng/mL Angiopoietin‐1 (ANG‐1) (Biotechne, Cat# 923‐AN‐025). VEGF and Aprotinin were reconstituted in DPBS, whereas Angiopoietin‐1 was reconstituted in 1% weight/volume Bovine Serum Albumin (BSA). Note that the included fetal bovine serum and gentamycin were excluded from the LifeFactors supplement kits. Medium was supplemented with heat‐inactivated Fetal Bovine Serum (Merck, F4135‐500ML) to 10% volume/volume. All media was filtered through sterile 0.22 µm pore filters before use.

### 4.3 Cell/Tissue Culture

Human placental pericytes (See Table S2) were cultured on 75 cm^2^ Easy Flasks (Thermo Fisher, Cat# 156499) coated with Quick Coat solution (3 mL, Angio–Proteomie, Cat# cAP‐01). Pericytes were cultured in Fibrolife medium, which is changed every other day. Pericytes were passaged up to three times prior to acoustic manipulation. GFP, RFP, and non‐reporter line human cardiac endothelial cells (See Table S2) were cultured on 75 cm^2^ Easy Flasks (Thermo Fisher, Cat# 156499) coated with Quick Coat solution (3 mL, Angio‐Proteomie, Cat# cAP‐01). The cells were cultured in the VascuLife medium with media changes occurring every other day. Fibroblasts (See Table S2) were cultured on 75 cm^2^ Easy Flasks (Thermo Fisher, Cat# 156499) in Fibrolife Medium. Fibroblast cultures did not require pre‐coating of flasks. Cells were passaged or used in experiments at >70% confluence by incubating cells in 0.05% Trypsin (Merck, Cat#T2601‐100ML) for 5–10 min at 37°C. The cells were then resuspended in FibroLife or VascuLife medium and centrifuged at 1000 RPM for 5 min. The cells were passaged up to two times prior to acoustic manipulation.

### 4.4 Bioink Formulation and Acoustic Manipulation

On the day of acoustic manipulation, a 5.0 mg/mL fibrinogen solution (Sigma‐Aldrich, Cat# F3879, St.) was prepared in DPBS (Calcium and Magnesium free, Thermo Fisher, Cat# 14190144, Waltham, MA) to encapsulate cells during alignment. A solution of thrombin (Sigma–Aldrich, Cat# T4393‐100UN) was prepared at 2.5 U/mL in parallel, and added in a 1:10 dilution to the fibrinogen solution to begin polymerizing the bioink (final thrombin concentration was 0.25 U/mL). The tissues would then be put on the acoustic platform and subjected to a sinusoidal wave with a frequency of 1.66 MHz, for at least 7 min at 30 Vpp. At 10 min, the slide was removed. The bioink would polymerize to the point of maintaining the acoustic patterning after 7 min, and would have vascular tissue medium added after 30 min to prevent partially polymerized portions of the gel from swelling and/or warping.

Fibroblasts, endothelial cells, and pericytes were dissociated from cell culture flasks as described previously. Cells were counted and resuspended at 1.5 million total cells per mL in DPBS, with a ratio of 1:1:0.1 for the fibroblasts: endothelial cells: and pericytes respectively. The three cell types were combined according to this density/ratio and centrifuged at 1000 RPM for 5 min, followed by aspiration of the supernatant and resuspension in the 5.0 mg/mL fibrinogen solution.

After the mixture was prepared. 270 µL of 5 mg/mL fibrinogen solution was added to a 30 µL aliquot of 2.5 U/mL thrombin and mixed vigorously. The sample was then loaded into two adjacent wells of an Ibidi 8‐well chamber slide, which was then placed in the central slot of the acoustic frame. Samples were then subjected to standing acoustic waves at 1.66 MHz for 7 min. Afterwards, samples were allowed to rest at room temperature for a minimum of 30 min before receiving 400 µL /well of Vascular Tissue medium. The samples were then placed in a 37°C, 5% CO_2_ incubator, where they had subsequent medium changes every other day. This would continue until day 7 when the samples would be fixed or disassociated for further study. Piezo temperature was measured with an infrared thermometer (Helect, 0.1°C resolution and ±2.7°C accuracy). The chambers did not have a temperature above 22°C during acoustic manipulation.

### 4.5 Microscopy

Time‐lapse images of GFP‐labeled endothelial cells were taken by an EVOS Auto FL (M5000, Thermo Fisher) on the days specified in the figure legends. Confocal immunofluorescent images were taken by using Leica TCS‐SP5 (Leica), TCS‐SP8 (Leica), as well as the Leica Stellaris (Leica). 3D and orthogonal reconstructed images were generated from images captured on the SP5 confocal microscope and processed in the LASX application suite (Leica).

For volumetric imaging, a Zeiss LSM 780 confocal microscope with a 40x/NA 1.2 water C‐Apochromat objective (Carl Zeiss) and a 488 nm Argon laser was used. The raw image data was deconvolved by using Huygens Professional (SVI).

### 4.6 Image Analysis

A 10X objective brightfield image was taken from each tissue (with and without acoustic manipulation) at day 0. Three images were taken from each sample (including the middle regions for top, center, and bottom of each chamber) for tissues without acoustic manipulation, and twelve images from the tissues acoustically manipulated to capture the variability in band uniformity. Two biological replicates were used as a comparison (without acoustic manipulation, n = 11), and four biological replicates (n = 16) were used to evaluate the acoustic manipulation effect. The images were processed in ImageJ to determine cell density, spacing, and the quality score. First, the image was segmented (using WEKA) with a classifier to separate the cells from empty spaces. The threshold was set to zero, divided by 255, and a glow Look‐Up Table (LUT) was applied for visualization. A script was developed to partition the image into bands. The signal area was measured, allowing for the calculation of a percentage area representing the cells. Band location was manually assigned, and pixel changes were set to increments of 50 in width (30.7 microns). To identify the best fit of the band location for the image, an 80:20 rule was applied to calculate a rating based on the Percentage of the Band Filled (PBF, with 0.2 weight) and the Percentage of Cells in the Bands (PCB, with 0.8 weight). This produced the band and gap widths for the PBF, and Gap Spacing (GS). To assess the quality of alignment and conformity, a weighted equation was used that incorporated the percentage density of the total area occupied by cells, the PBF, and a compensation derived from the GS. Node spacing for the equation was set to 600 pixels (370 microns).

Quality of Alignment and Conformity Equation

(1) $$Density\times 0.25+PBF\times 0.75-\left(\frac{GS-N}{N}\right)\times 12$$

$$PCB=\frac{Area\;from\;Signal\;in\;the\;Band}{Total\;Area\;from\;Signal\;Image}$$

$$PBF=\frac{Area\;from\;Signal\;in\;the\;Band}{Band\;Area}$$

GS = Gap Spacing From Acoustically Manipulated Tissue

N = Node Spacing

Tissues were imaged on day 7 to assess the vasculature in ImageJ. The location of day 0 was matched to their day 7 counterparts. After image acquisition, image intensities were normalized. Two parameters were measured; percentage area and number of branches. For branches, the Ridge Detection tool was applied. Vessels less than 70 microns in length were excluded from the analysis. For the area, the images were processed with a minimum threshold of 55, and the percentage area occupied was measured from the binary data. Images with vasculature that extended beyond the focal field or poorly matched regions were excluded from the dataset.

For wide view tissue comparison between control (without acoustic manipulation) and with acoustic manipulation, images of GFP‐labeled endothelial cells with 4X objective were taken at day 7. Three images were taken per sample, corresponding to four paired biological samples (n = 4). Four parameters were measured; percentage area, number of branches, mean branch length, and maximum branch length (Figure S2). The evaluation was the same as mentioned above for the 10X images.

To compare methods, a Voronoi Tessellation evaluation was carried out in selected samples (Figure 2B, Figure S2). Brightfield images were analyzed in ImageJ. Cell points were selected manually, and the Voronoi plugin was used. A threshold of 1–255 was applied, and the image was converted into binary with the erode tool to emphasize borders (Figure S1A). The Analyze Particles tool was used to determine the area of the individual polygons and thus cell proximity. Median and maximum area of polygons are reported in Table S1.

Forty cells were manually measured using ImageJ oval and area tool from single‐cell culture and Full Tissue samples. Values were converted into diameter for comparison.

### 4.7 Tissue Fixation and Staining Methods

Fixation: Tissues were fixed in paraformaldehyde (4%) in 1x DPBS at 4°C overnight or at room temperature for 15 min for fixation and were washed three times for 10 min in 1x PBS. If whole tissue samples were to be stained directly, samples were permeabilized using in 0.5% Triton X‐100 (Thermo Fisher Scientific, Cat# 85112) solution in 1x PBS for 30 min at room temperature. Samples were carefully removed with a sterile metal spatula, transferred to the 24‐well, and blocked in 5% goat serum, 0.1% Triton X‐100, and 1%BSA in 1x PBS for 2 h at room temperature on an agitation plate. Afterward, the samples were washed three times for 10 min in 1x PBS prior to immunostaining. Primary stains (see Table S3 for used antibodies and probes) were incubated overnight at 4°C, then washed the following day three times for 10 min in 1x PBS. Secondary staining (see Table S3 for used antibodies and probes) then followed, where samples were incubated at room temperature for 4 h. Samples were then washed three times for 10 min in 1x PBS for the final time, before being mounted on slides for imaging.

### 4.8 Quantitative PCR

Tissue samples were cultured for a period of 7 days, then removed from their wells with a flat sterile spatula before being transferred to a 24‐well plate. Tissues of similar experimental groups were combined into a single well, with a minimum of 4 tissues being added. Tissues were then gently minced with a surgical scalpel in 4 mL of enzyme solution consisting of collagenase II (1.5 mg/mL, Thermo Fisher Scientific Cat# 17101‐015), dispase I (0.2 mg/mL, Sigma–Aldrich, Cat# D4818), DNase I (0.5 mg/mL, Worthington Biomedical Corp, Cat# LS002007) in DPBS. Tissues were incubated for 10 min in enzyme solution at 37°C.To stop enzymatic digestion and remove the enzyme, 10 mL of Vascular Tissue medium was added to each tube and filtered through the 20 µm cell strainer (MilliporeSigma, Cat# SCNY000200).

Cells were then lysed and underwent RNA isolation as detailed in the NucleoSpin RNA Plus XS RNA isolation kit (Fisher Scientific, Cat# 15751639). Afterwards, RNA content was quantified by measuring A_260_/A_280_ on a Nanodrop 2000c Spectrophotometer (Thermo Scientific, Cat # ND‐2000). cDNA libraries were generated from the resulting product, following the protocol details in the RevertAid First Strand cDNA Synthesis Kit (Thermo Fisher, Cat# K1621) and operated with a Personal Thermocycler (Biometra, Cat# 846‐070‐234). Finally, samples were loaded into the RT^2^ Profiler PCR Array Human Angiogenesis plate (Qiagen, Cat# 330231), and run on the QuantStudio 6k Flex Real‐Time PCR system (Thermo Fisher, Cat # 4485689) as specified in the RT^2^ Profiler PCR Array Human Angiogenesis instruction guide. Results were then analyzed on QuantStudio Software v1.3. Gene expression was normalized against the following housekeeping genes: Actin‐Beta (ACTN), Beta‐2‐microglobulin (B2M), Glyceraldehyde‐3‐Phosphate dehydrogenase (GAPDH). Hypoxanthine phosphoribosyltransferase 1(HPRT1), and Ribosomal protein, large, P0 (RPLP0). Gene expression when compared against unaligned endothelial cell tissue samples, with the resulting fold change being relevant to said samples

### 4.9 Statistical Analysis

Biological “n” is an independent biological replicate in each figure. We determined our biological replicates to be unique combinations of cells derived from varied donors and vendor lot numbers. For example, a biological replicate would have fibroblasts from lot 510Z036.3, endothelial cells from lot 2023050302, and pericytes from 489z002.2. Tissues which contained similar cell backgrounds with only a variation in the monocyte donor would not be considered a separate biological replicate. Measurements taken from these tissues would be recorded initially as technical replicates, then averaged to provide a mean value, which constituted a value reflecting the biological replicate corresponding to those samples with conserved cell sources. Biological vs. Technical n are clearly identified in their respective figures. Two Way ANOVA, with Tukey's and Sidak's multiple Comparisons Test was performed for gene expression analysis. Results were derived from a biological n of 4 with normal data distribution in accordance with biological data reporting standards (full details may be found in the data repository). Statistical comparison for EC with and without acoustics with less than 0.8 power was as follows; *p* values for CDH5 *p* = 0.012, EGF *p* < 0.001, KDR *p* = 0.022, CXCL1 *p* < 0.005, PECAM1 *p* < 0.005, VEGF‐b *p* = 0.019, and power = 0.51, 0.69, 0.70, 0.21, 0.61, and 0.69, respectively. For Full Tissues with and without acoustics with less than 0.8 power was as follows; Collagen 18A1 *p*  =  0.007, EGF *p* < 0.005, KDR *p* = 0.039, LEP *p* = 0.019, and PECAM1 *p* = 0.019, and power = 0.62, 0.46, 0.40, 0.67, and 0.70, respectively. Paired t‐test was used to find mean significant differences between two conditions (with and without acoustic manipulation), such statistical tests were two‐tailed. For failed normality tests (Shapiro–Wilk) on wide tissue comparison (4X data), maximum branch length and area were further analyzed with Wilcoxon, resulting in *p* < 0.001 and *p*  =  0.48, respectively. The minimum power sample where a statistical difference (*p* > 0.05) was reported for analysis was 0.78. All statistical analyses were performed using Graphpad Prism version 8.2.0 for Windows (GraphPad Software, San Diego, CA), Microsoft Excel for Microsoft 365 MSO (16.0.13001.20266) 32‐bit (Microsoft, Redmond, WA), or R (RStudio, Version 2024.12.1, Posit). *p*‐value < 0.05 was considered significant. Each statistical analysis method was indicated in individual quantification sections.

## Author Contributions

O. O. L. J., R. Q., and D. F. D. C. conceived and designed the study. R. Q., O. O. L. J., and K. P. developed the methodology. The investigation was carried out by R. Q., O. O. L. J., K. P., N. K. R., and A. F. Data visualization was performed by R. Q., O. O. L. J. and D. F. D. C. D. F D. C. supervised the project. R. Q. and O. O. L. J. prepared the original manuscript draft. R. Q., O. O. L. J., D. F. D. C., N. K. R., K. P., and A. F. contributed to reviewing and editing the manuscript and approved the final version.

## Funding

This project has received funding from the European Research Council (ERC) under the European Union's Horizon 2020 research and innovation programme (Grant agreement No. 101077419 – LIGHTHEART) to DFDC. This work was supported by the Deutsche Forschungsgemeinschaft (DFG, German Research Foundation) under Germany's Excellence Strategy via the Excellence Cluster 3D Matter Made to Order (Grant No. EXC‐2082/1–390761711) to DFDC. This Work Was Supported by the Health + Life Science Alliance Heidelberg Mannheim and received state funds approved by the State Parliament of Baden‐Württemberg (Grant STRETCH‐HEART) to DFDC, OOLJ, and RQ.

## Conflicts of Interest

The authors declare no conflicts of interest.

## Supporting information




**Supporting File 1**: advs76219‐sup‐0001‐SuppMat.docx.

**Supporting File 2**: advs76219‐sup‐0002‐MovieS1.MP4.

**Supporting File 3**: advs76219‐sup‐0003‐MovieS2.avi.

**Supporting File 4**: advs76219‐sup‐0004‐MovieS3.avi.

## Data Availability

The data that support the findings of this study are available from the corresponding author upon reasonable request.
