# Supplementary material for: Quality Thresholds for Angiogenesis Under Acoustic Manipulation in Engineered Vascular Tissues
Source: Adv Sci (Weinh). 2026 Jun 23:e76219. Online ahead of print. doi: 10.1002/advs.76219 (PMC13336421; doi:10.1002/advs.76219)
Supplement: Supplementary file 1 — Supporting File 1: advs76219‐sup‐0001‐SuppMat.docx. [file ADVS-9999-e76219-s004.docx]

Supplementary Materials for

**Quality thresholds for angiogenesis under acoustic manipulation in engineered vascular tissues**

Oscar O’Dwyer Lancaster-Jones^1^ † *, Russell Quinn^1^ † *, Niloofar Khoshdel Rad^1^, Kilian Paul^1^, Andrea Frank^1^, Daniela F. Duarte Campos^1^*

^1^Bioprinting and Tissue Engineering Group, ZMBH, Heidelberg University

†These authors contributed equally to this work and are listed alphabetically as co-first authors

*Correspondence to: Oscar O’Dwyer Lancaster-Jones, [o.odwyer@zmbh.uni-heidelberg.de](mailto:o.odwyer@zmbh.uni-heidelberg.de); Russell Quinn, [qruss91@gmail.com](mailto:qruss91@gmail.com); and Daniela Duarte Campos, [dcampos@uni-heidelberg.de](mailto:dcampos@uni-heidelberg.de)

**This PDF file includes:**

Figs. S1 to S5

Tables S1 to S3

Movies S1 to S3


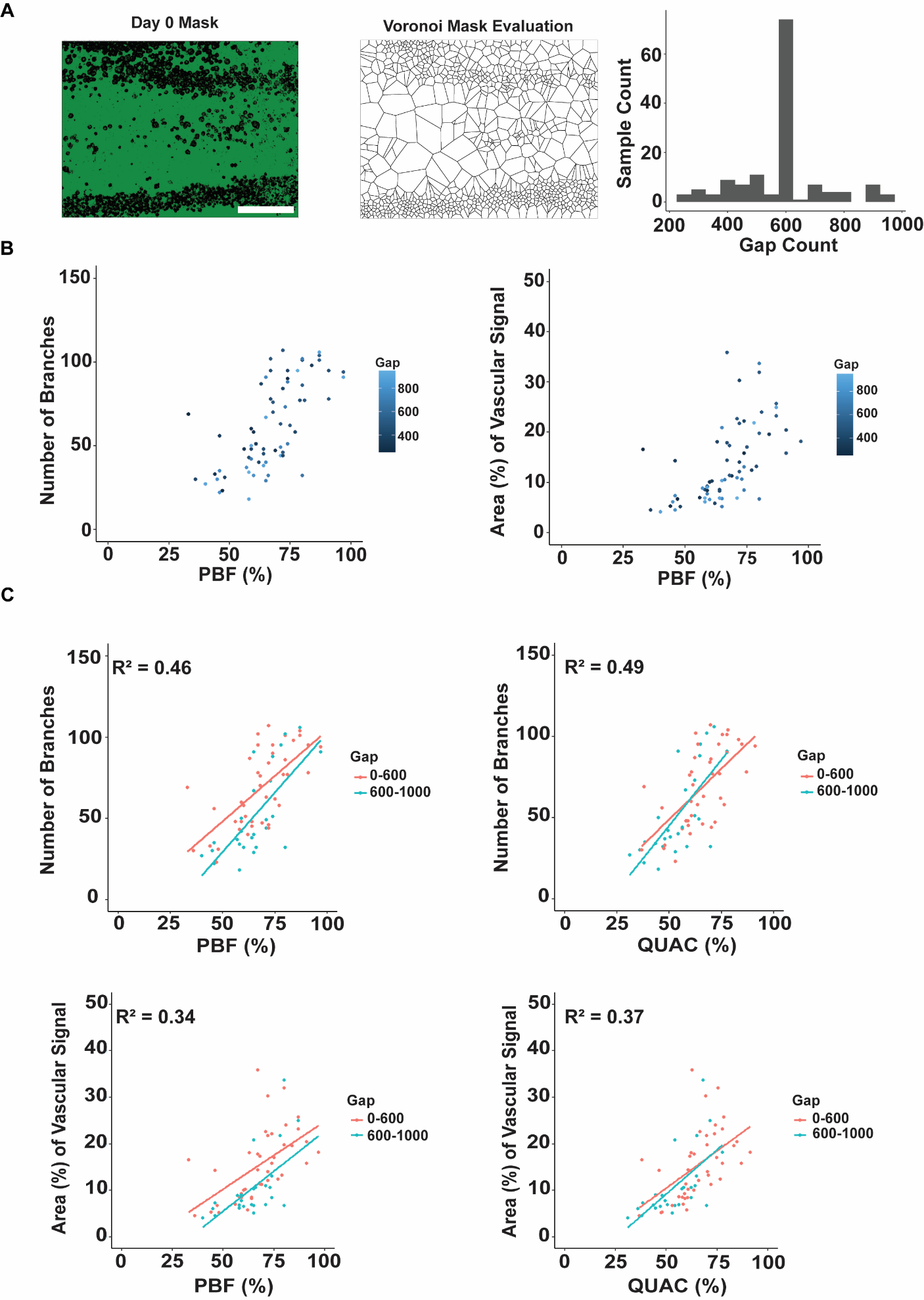


Fig. S1. Voronoi Tessellation Evaluation and Gap Correlations of Tissues With Acoustic Manipulation.

**A,** Processed image for density and band analysis, left, and counterpart Voronoi Tessellation mask after processing from the brightfield image (10X), middle. Gap spacing (pixels) histogram for acoustically manipulated tissues (n=141), right. **B,** Scatterplots of percentage area from vasculature and number of branches from acoustically manipulated tissues where gap spacing was not 600 pixels (approx. 370 microns) against percentage of the band filled (n=67). **C,** Scatterplots of percentage area from vasculature and number of branches from acoustically manipulated tissues where gap spacing was smaller than 600 pixels in red (n=41) and larger than 600 pixels in blue (n=26), against percentage of band filled and quality of alignment and conformity. R^2^ reported for pooled dataset. Dataset excludes values with a gap of 600 pixels.


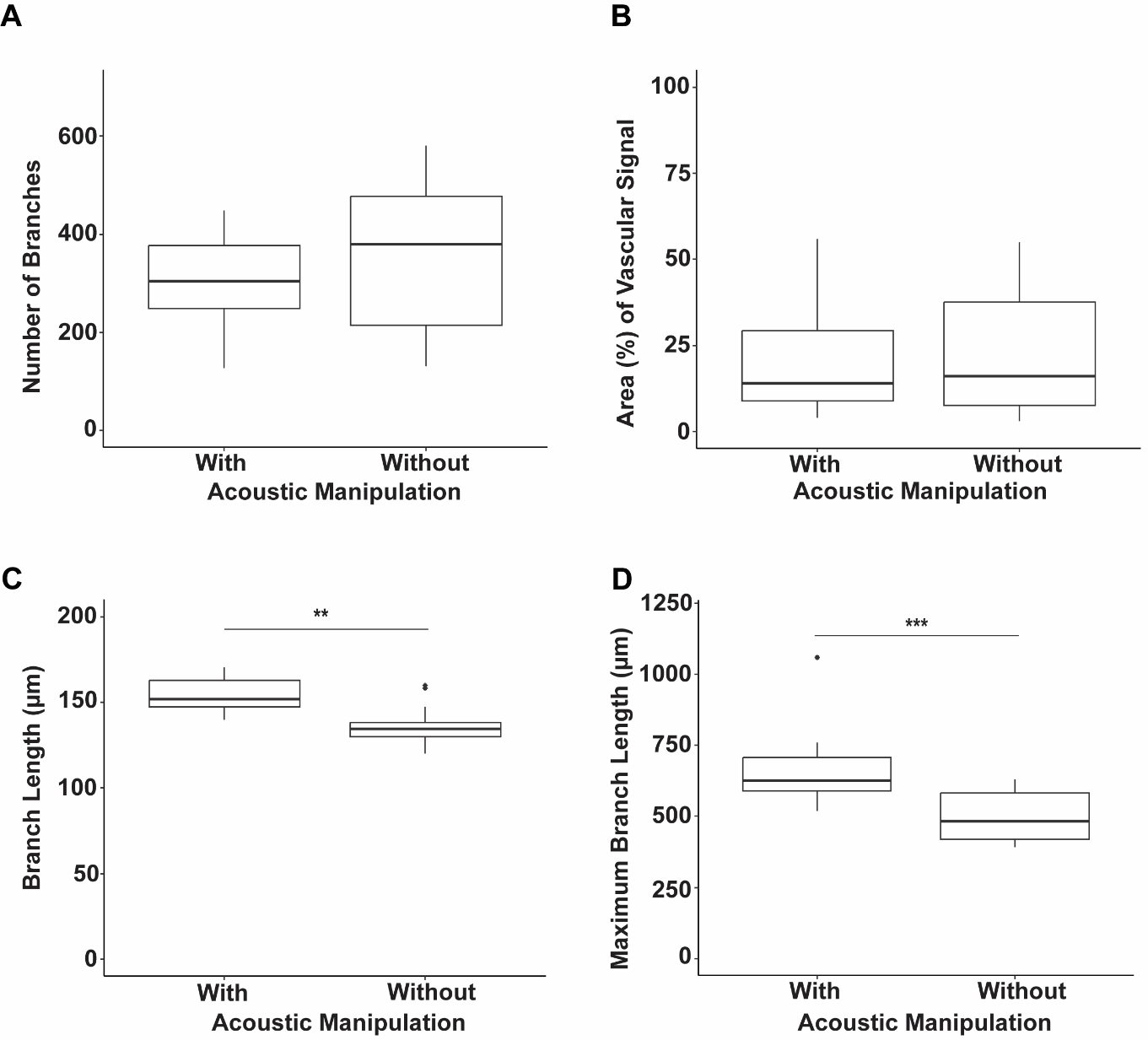


Fig. S2. Quantification of Vascular Profiles in Tissues With and Without Acoustic Manipulation.

**A**, Measurements for number of branch points in Full Tissues with and without acoustic manipulation. Quantifications are based on 4X objective images from an EVOS microscope. Twelve images per group were analysed with two-tailed paired t test, p = 0.26. Samples came from biological replicates with an n = 4. **B**, Measurements signal area percentage in Full Tissues with and without acoustic manipulation. Quantifications are based on 4X objective images from an EVOS microscope. Twelve images per group were analysed with two-tailed paired t test, p =0.73 Samples came from biological replicates with an n = 4. **C**, Measurements for branch length in Full Tissues with and without acoustic manipulation. Quantifications are based on 4X objective images from an EVOS microscope. Twelve images per group were analysed with two-tailed paired t test, p = 0.008. Samples came from biological replicates with an n = 4. **D**, Measurements for maximum branch length in Full Tissues with and without acoustic manipulation. Quantifications are based on 4X objective images from an EVOS microscope. Twelve images per group were analysed with two-tailed paired t test, p = 0.001. Samples came from biological replicates with an n = 4.


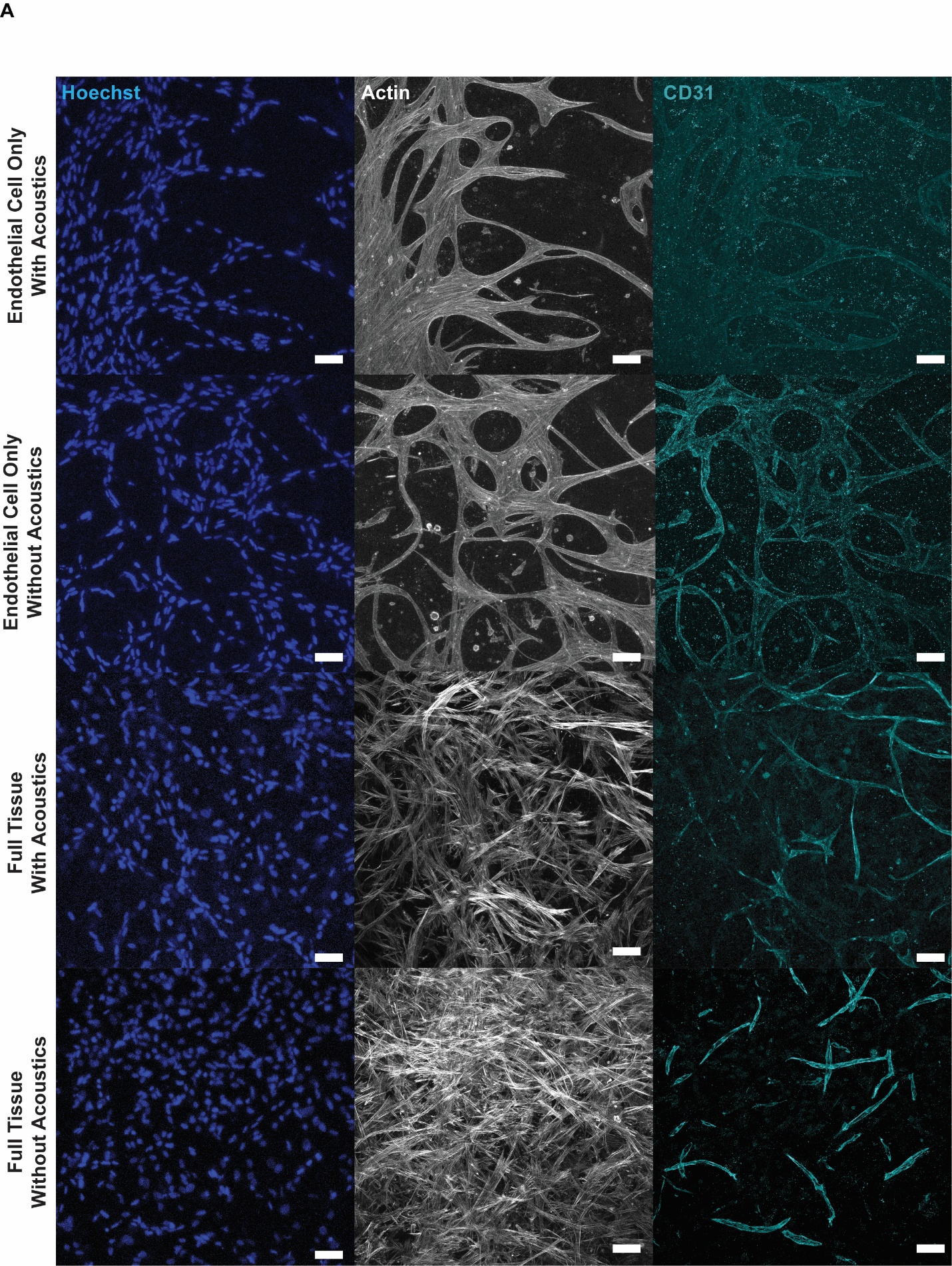


Fig. S3. Organization of Actin-Cytoskeletal Space for Co-Cultured and Endothelial Cell Only Tissues.

**A,** Maximum intensity projection immunostaining images for actin cytoskeleton markers (Phalloidin, white), endothelial cell markers (CD31, cyan), and Hoescht (blue) on flatmount samples of tissues with and without acoustic manipulation. Images were taken with a 10X objective on a Leica SP5 confocal microscope. Tissues were 7 days old at time of acquisition. Scalebar = 50 µm


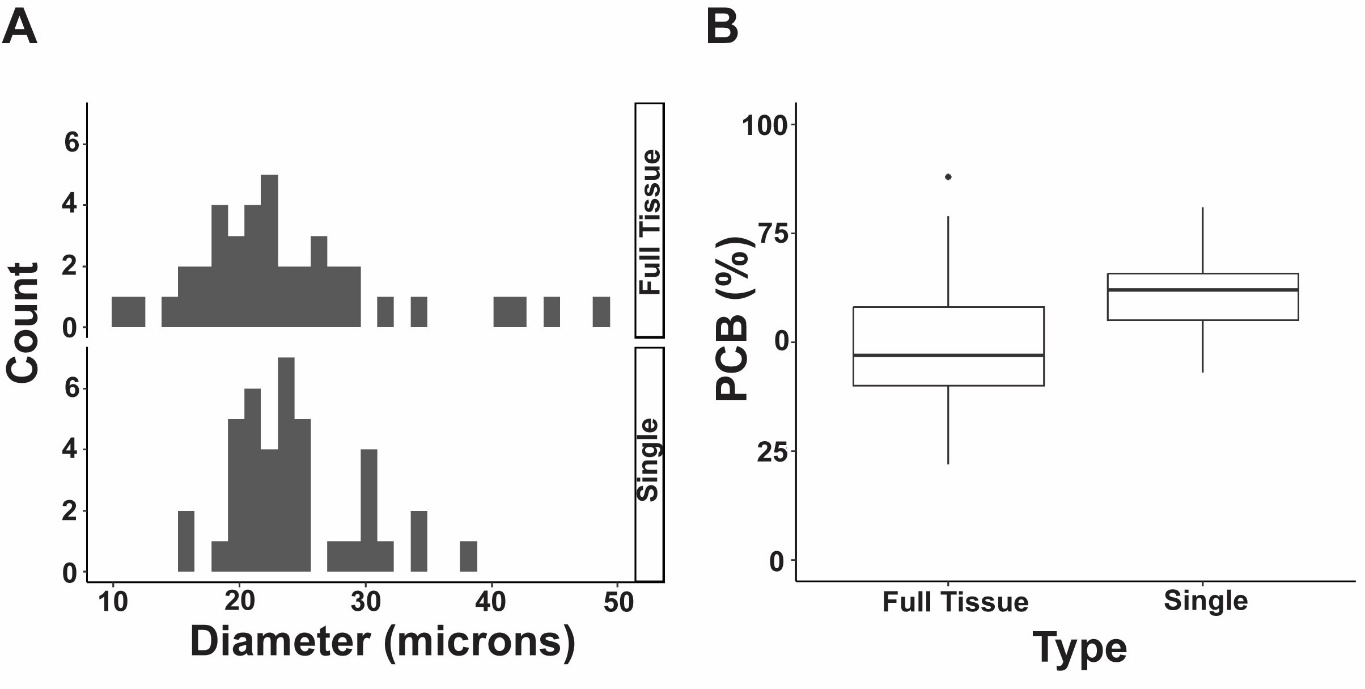


**Fig. S4. Cellular variation from single type and Full Tissues**

**A**, Frequency of cell diameter analysis using ImageJ from single cell culture versus Full Tissue in a non-acoustically manipulated sample after dispensing in the chamber. **B**, Percentage of cells in bands in acoustically manipulated tissues for single culture (n=18) versus Full Tissue (n=141).


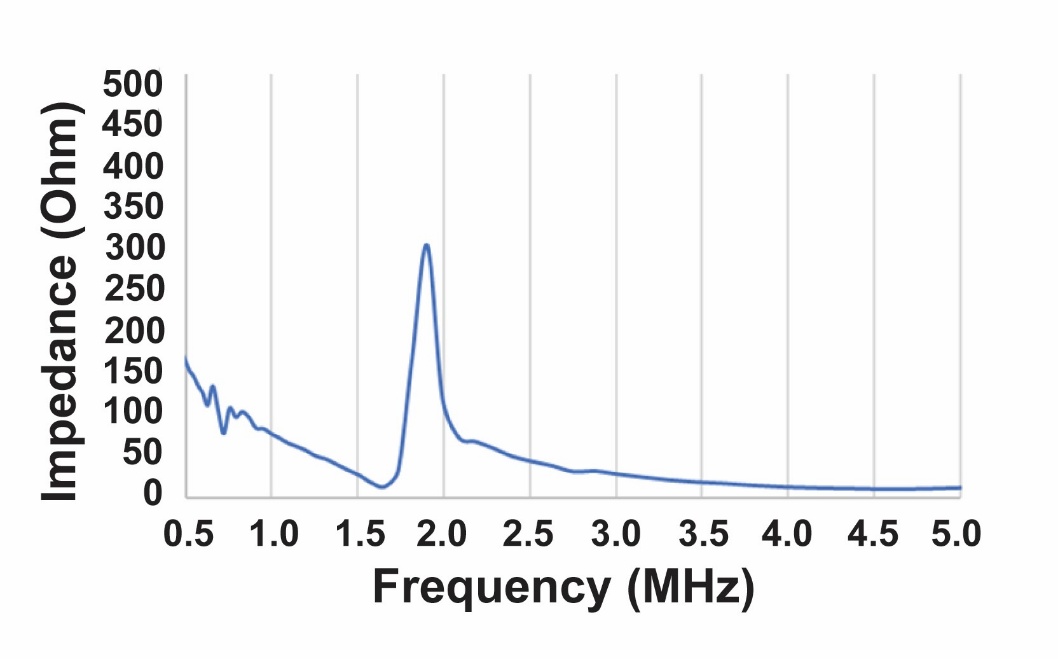


**Fig. S5. Frequency sweep versus impedance for M165D25 Midas piezo attached to 3D printed holder with water bath.**

Table S1. Evaluation Across Different Methodologies of Acoustically Manipulated Vascular Tissues

Data for four 10X image samples providing the Percentage Band Filled (PBF), Quality of Alignment and Conformity (QUAC) and area (median and maximum values) from Voronoi evaluation against vasculature evaluation.

| Sample | 1 | 2 | 3 | 4 |
| --- | --- | --- | --- | --- |
| PBF | 46 | 64 | 59 | 74 |
| QUAC | 39 | 51 | 54 | 74 |
|  |  |  |  |  |
| Voronoi Area Evaluation (pixels) | | | | |
| Median | 1299 | 1175 | 1573 | 1256 |
| Maximum | 45000 | 85000 | 90000 | 80000 |
|  |  |  |  |  |
| Vasculature Evaluation | | | | |
| Branches (n) | 35 | 38 | 34 | 95 |
| Area (%) | 7.3 | 6.8 | 7.7 | 22.2 |

Table S2. Cell Sources.

List of cells, vendors, and identification information used in construction of tissues.

| Cell Type | Catalog Number | Lot Number | Vendor |
| --- | --- | --- | --- |
| Human Cardiac Coronary Artery Endothelial Cells | FC-0032 | N/A | Cell Systems |
| Human Cardiac Coronary Artery Endothelial Cells (RFP) | PB-cAP-0007RFP | N/A | PELO Biotech |
| Human Cardiac Coronary Artery Endothelial Cells (GFP) | cAP-006GFP | 2023050302 | PELO Biotech |
| Human Cardiac Coronary Artery Endothelial Cells (GFP) | cAP-006GFP | 2025032602 | PELO Biotech |
| Human Aortic Artery Endothelial Cells (GFP) | cAP-007GFP | 2023050302 | PELO Biotech |
| Human Cardiac Coronary Artery Endothelial Cells (GFP) | cAP-006GFP | 2025032602 | PELO Biotech |
| Human Placental Pericytes | C-12979 | 489z002.1 | Promocell |
| Human Placental Pericytes | C-12980 | 489z002.2 | Promocell |
| Human Placental Pericytes | C-12980 | 489z020 | Promocell |
| Human Cardiac Fibroblasts | C-12375 | 495z037.1 | Merck |
| Human Cardiac Fibroblasts | C-12375 | 510Z036.3 | Merck |
| Human Cardiac Fibroblasts | C-12376 | 494Z043.6 | Merck |

**Table S3. Utilized Antibodies.**

List of antibodies, dilutions, vendors, and identification information used in construction of tissues.

| Antibody | Dilution | Vendor | | Cat # |
| --- | --- | --- | --- | --- |
| Antibody | **Dilution** | **Catalog Number** | **Vendor** | |
| CD31 (PECAM) | 1 to 100 | MO82329-2 | Aligent | |
| Collagen Type I | 1 to 100 | 14695-1-AP | Proteintech | |
| Nestin | 1 to 100 | AB18102 | Abcam | |
| NG2 | 1 to 100 | AB279348 | Abcam | |
| PDGFR-Alpha | 1 to 100 | AB96569 | Abcam | |
| VEGFA | 1 to 100 | 66828-1-lg | Proteintech | |
| Vimentin | 1 to 100 | AB137321 | Abcam | |
| Goat Anti-Mouse A488 | 1 to 1000 | A32723 | Life Technologies | |
| Goat Anti-Mouse A555 | 1 to 1000 | A32727 | Life Technologies | |
| Goat Anti-Mouse A647 | 1 to 1000 | A32728 | Life Technologies | |
| Goat Anti-Rabbit A488 | 1 to 1000 | A32731 | Life Technologies | |
| Goat Anti-Rabbit A555 | 1 to 1000 | A32732 | Life Technologies | |
| Goat Anti-Rabbit A647 | 1 to 1000 | A32733 | Life Technologies | |
| Hoescht 33342 | 1 to 2000 | 62249 | Fisher Scientific | |
| Phalloidin A488 | 1 to 300 | A12379 | ThermoFisher | |
| Phalloidin A594 | 1 to 300 | A12381 | ThermoFisher | |

Movie S1.

Brightfield microscopy video demonstrating the acoustic manipulation of cells in fibrin hydrogel in real time. Video was recorded on iPhone 16, utilizing a 2X magnification on a Leica M80 stereo microscope.

**Movie S2.**

3D reconstruction of acoustically manipulated vascular tissue. Samples were stained for Collagen type-I (COL-I, magenta), Vascular Endothelial Growth Factor A (VEGF-A, cyan), and Endothelial Cell GFP reporter (GFP, white) on acoustically manipulated Full Tissues. Images were taken with a 63X objective on a Leica SP8 confocal microscope, and 3D reconstruction was generated in the Leica X processing Suite.

**Movie S3.**

3D reconstruction of acoustically manipulated endothelial cell only tissue. Samples were stained for Collagen type-I (COL-I, magenta), Vascular Endothelial Growth Factor A (VEGF-A, cyan), and Endothelial Cell GFP reporter (GFP, white) on acoustically manipulated endothelial cell only tissues. Images were taken with a 63X objective on a Leica SP8 confocal microscope, and 3D reconstruction was generated in the Leica X processing Suite.
